# Cannabinoid Receptors CB1 and CB2 Activation Restores Hippocampal Lipid Profiles and Alleviates Autism‐Like Behaviors in Valproic Acid‐Induced ASD Rats

**DOI:** 10.1111/cns.70591

**Published:** 2025-08-25

**Authors:** Haoran Wang, Mengyuan Zhang, Sen Yang, Yi Jiang, Lijie Wu, Caihong Sun

**Affiliations:** ^1^ Department of Children's and Adolescent Health, Public Health College Harbin Medical University Harbin China; ^2^ Heilongjiang Province Key Laboratory of Child Development and Genetic Research Harbin Medical University Harbin China; ^3^ Department of Developmental Behavioral Pediatrics The Sixth Affiliated Hospital of Harbin Medical University Harbin China

**Keywords:** autism spectrum disorders, cannabinoid receptor 1, cannabinoid receptor 2, lipidomics

## Abstract

**Objective:**

Emerging evidence suggests lipid metabolism dysregulation contributes to autism spectrum disorders (ASD), with the endocannabinoid system (cannabinoid receptors CB1R/CB2R) implicated in lipid homeostasis. This study investigated whether CB1R/CB2R activation improves hippocampal lipid metabolism and ASD‐like behaviors in a valproic acid (VPA)‐induced ASD rat model.

**Methods:**

Male offspring from dams exposed to VPA (600 mg/kg, i.p.) received the CB1R agonist ACPA (0.1 mg/kg) or the CB2R agonist AM1241 (3 mg/kg) from postnatal days 21–27. ASD‐like behaviors (marble burying, self‐grooming, social interaction, open‐field tests) and hippocampal lipid profiles (UPLC‐MS/MS) were analyzed.

**Results:**

VPA‐exposed rats displayed heightened repetitive behaviors, social deficits, and hyperactivity, all significantly alleviated by ACPA and AM1241. Lipidomics revealed marked reductions in hippocampal phosphatidylcholines, lysophosphatidylcholines, fatty acids, sphingomyelins, ceramides, and phosphatidylethanolamines in VPA rats. Both agonists restored lipid levels to near normal, comparable to controls.

**Conclusions:**

CB1R/CB2R activation ameliorates behavioral abnormalities and rectifies hippocampal lipid dysregulation in VPA‐induced ASD models, highlighting cannabinoid receptors as potential therapeutic targets for ASD‐associated metabolic disturbances.

## Graphical abstract

CB1R/CB2R activation ameliorates behavioral deficits and rectifies hippocampal lipid dysregulation in a VPA‐induced ASD rat model, highlighting the endocannabinoid system's therapeutic potential for autism spectrum disorders.

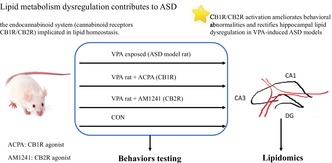

## 1 Introduction

Autism spectrum disorders (ASD) are complex neurodevelopmental conditions characterized primarily by social communication deficits and repetitive, stereotyped behaviors [1]. Over the years, the prevalence of ASD has notably increased, with the latest estimates indicating that about 2.78% of children are affected [2]. Despite extensive research, the etiology and underlying pathogenesis of ASD remain incompletely understood. Emerging evidence suggested that abnormalities in lipid metabolism may influence energy biosynthesis, signal transduction, oxidative stress, and inflammation, thereby might be involved in ASD pathophysiology [3]. Several recent metabolomics studies demonstrated altered plasma levels of phosphatidylcholine, multiple acylcarnitines, and significant disturbances in glycerophospholipid and glyceryl ester metabolism in children with ASD [4, 5]. Consistently, our research team has previously identified aberrant serum lipid metabolism in a cohort of ASD patients compared to healthy individuals [6]. What's more, longitudinal cohort studies have shown that levels of lysophosphatidylcholines (LPCs), phosphatidylethanolamines (PEs), sphingomyelins (SM) and ratios of nonesterified fatty acids at birth were significantly associated with the later severity of ASD symptoms [7, 8]. These findings collectively supported a significant link between lipid imbalances and ASD.

The endocannabinoid system (ECS), consisting of G protein‐coupled cannabinoid receptors (primarily cannabinoid receptor 1 [CB1R] and cannabinoid receptor 2 [CB2R]) and their endogenous lipid‐derived agonists, is closely related to regulating lipid metabolism. This includes fatty acid (FA) metabolism, triglyceride, lipoprotein levels, and cholesterol homeostasis [9, 10, 11]. Previous studies have highlighted critical roles for CB1R and CB2R in modulating lipid accumulation and lipid profile expression across various tissues, suggesting their potential involvement in ASD‐associated metabolic dysfunctions. Additionally, our research group has identified abnormal expression patterns of CB1R and CB2R in both children with ASD and ASD model rats, further correlating these abnormalities with ASD‐like behavioral phenotypes [12]. However, the precise mechanisms by which CB1R and CB2R signaling contribute to lipid dysregulation in ASD remain unknown.

As lipidomics emerges as a powerful tool in metabolomics, it has gained increasing attention in revealing the relationship between lipid metabolites and diseases, such as ASD. To date, however, no studies have explored the direct relationship between CB1R/CB2R signaling and lipid metabolism abnormalities in ASD. To address this gap, the current study employed a valproic acid (VPA)‐induced rat model of ASD to investigate how activation of CB1R (agonist ACPA) and CB2R (agonist AM1241) impacts hippocampal lipid metabolism using targeted lipidomics. Furthermore, we evaluated behavioral changes in VPA‐exposed rats following CB1R and CB2R activation to assess whether modulation of the ECS can alleviate ASD‐like behaviors. Our findings offer new insights into the mechanistic link between cannabinoid receptor signaling and lipid metabolism in ASD, potentially identifying novel therapeutic targets for future interventions.

## 2 Methods

### 2.1 Animals and Treatments

Adult male and female Wistar rats (aged 7–8 weeks) were obtained from Bei Jing Weitong Lihua Biotechnology Co. Each rat was housed individually in a controlled setting with a stable temperature (22°C ± 2°C), humidity (50% ± 10%), and a 12‐h day‐night cycle. Rats had unlimited access to drinking water and standard rodent feed. The experimental process for inducing the animal model of ASD using VPA exposure was performed following a previously described protocol [12]. Briefly, female and male rats mated overnight. Pregnancy was verified the following morning through the detection of vaginal plugs, defining midday of that day as embryonic day 0.5 (E0.5). On E12.5, pregnant females received a single intraperitoneal (i.p.) injection of VPA (Sigma Aldrich, St. Louis, MO, USA) at 600 mg/kg between 08:00 and 09:00. VPA was prepared at 250 mg/mL in 0.9% saline. Control animals were administered saline only. Pregnant rats were housed separately and allowed to care for their offspring. Offspring were weaned on postnatal day (PND) 21, housed 4–5 per cage, and male offspring were chosen for experiments to prevent variability due to female estrous cycles.

Two cannabinoid receptor agonists, APCA (CB1R agonist; MedChemExpress, Cat No.: 229021–64‐1) and AM1241 (CB2R agonist; Selleck, Cat No.: S154401), were dissolved initially in DMSO at 50 mg/mL. This stock solution was then further diluted with Tween 80, PEG400, and distilled water in a ratio of 1:8:10 to achieve a final concentration of 2.5 mg/mL. Male offspring received intraperitoneal injections twice daily from PND21 to PND27: APCA at 0.1 mg/kg, AM1241 at 3 mg/kg, or vehicle alone. Dosages were determined based on earlier research findings [13, 14, 15].

The male offspring were randomly allocated into four experimental groups: (1) CON (control rats injected with vehicle); (2) VPA (VPA rats injected with vehicle solution); (3) ACPA (VPA rats injected with 0.1 mg/kg APCA); (4) AM1241 (VPA rats injected with 3 mg/kg AM1241). After treatment completion, randomly selected rats from each group (*N* = 10–12 per group) underwent behavioral assessments before euthanasia by decapitation for lipidomic analyses.

### 2.2 Behavioral Testing

Behavioral testing was conducted sequentially in the following order: open field, marble burying, self‐grooming, and social interaction tests, arranged from least to most stressful. A 15‐min interval was maintained between each test to minimize potential carry‐over effects. As described previously [12], behavioral testing was recorded using video cameras and analyzed with the SMART software (version 3.0, Panlab, Barcelona, Spain). Testing was conducted from 09:00 to 17:00, and equipment was cleaned with 75% ethanol between each session to avoid scent cues. Observers analyzing behaviors were blinded to the experimental conditions.

#### 2.2.1 Open Field Test

Hyperactivity and anxiety‐related behavior were examined in a gray Plexiglas open field box (45 × 45 × 40 cm). Each rat explored freely for 5 min before being placed in the center for a 10‐min evaluation period. The total distance movement and resting periods were recorded.

#### 2.2.2 Marble Burying Test

This test was used to assess repetitive and stereotyped behavior. The rats were placed individually in a cage containing a 5 cm layer of fresh bedding material, allowing 15 min for acclimation. Afterward, 20 glass marbles (12 mm diameter) arranged evenly in four rows were placed in the cage, and rats were observed for 30 min. Marbles buried at least two‐thirds deep were counted.

#### 2.2.3 Self‐Grooming Test

Spontaneous grooming behavior was recorded for 10 min after a 5‐min habituation period in individual cages (48 × 35 × 20 cm) and allowed to habituate for 5 min, followed by the recording of self‐grooming for 10 min. Grooming activities measured included paw licking, unilateral and bilateral strokes around the nose, mouth, and face, paw movement over the head and behind the ears, body fur licking, body scratching with hind paws, tail licking, and genital cleaning.

#### 2.2.4 Social Interaction Test

Before testing, each rat was isolated individually for one night. Following a 5‐min acclimation in a test cage (45 × 45 × 60 cm) filled with fresh bedding, an unfamiliar rat matched for sex and age was introduced for a 15‐min interaction. Playful social interactions (pouncing, pinning, social exploration) and nonsocial behaviors (social evasion, inaction, self‐grooming) were recorded [16].

### 2.3 Targeted Lipidomics Analysis

#### 2.3.1 Tissue Preparation

Hippocampal tissue samples were collected, immediately snap‐frozen using liquid nitrogen, and preserved at −80°C until further processing. Hippocampus tissues (50 mg) were weighed into a 2 mL centrifuge tube and homogenized for 1 min. 300 μL methanol and 1 mL methyl tert‐butyl ether (MTBE) were added to the tube, and the mixture was vortexed for 1 h. Then, the samples were added to 250 μL water and vortexed for 10 s before being centrifuged for 10 min at 12,000 rpm at 4°C. The supernatant (95 μL) and isotope standard solution (5 μL) were charged into a 1.5 mL centrifuge tube and vortexed for 2 min. Subsequently, the mixture was transferred into a vial for mass spectrometry (MS) detection.

#### 2.3.2 Chromatographic Conditions

Chromatographic separation was conducted on a Waters ACQUITY I‐CLASS ultra‐performance liquid chromatograph (UPLC) system equipped with a Waters UPLC BEH C8 column (100 mm × 2.1 mm, 1.7 μm). In positive ion mode, the mobile phase consisted of phase A (acetonitrile/water at a ratio 6:4, v/v, supplemented with 5 mM ammonium formate and 0.1% formic acid) and phase B (isopropanol/acetonitrile at a ratio of 9:1, v/v, containing 5 mM ammonium formate and 0.1% formic acid). The flow rate was set at 0.26 mL/min and the column temperature was maintained at 55°C. Gradient elution conditions are detailed in Table S1, and a sample injection volume of 5.0 μL was employed. For negative ion mode, the mobile phase included phase A (acetonitrile/water at a ratio of 1:10, v/v, containing 1 mM ammonium acetate and 0.04% formic acid) and phase B (isopropanol/acetonitrile at a ratio of 1:1, v/v). Here, the flow rate was adjusted to 0.3 mL/min with the same column temperature of 55°C. Gradient elution conditions are provided in Table S1, and the injection volume was also 5.0 μL.

#### 2.3.3 Mass Spectrometry Conditions

Mass spectrometry (MS) analyses in both positive and negative ion modes were carried out using a WATERS XEVO TQ‐S MICRO system. Instrumental parameters included ion source voltage at 3.0 kV, ion source temperature at 150°C, desolvation temperature set to 350°C, desolvation gas flow rate of 1000 L/h, cone voltage at 29 V, and cone gas flow at 150 L/h.

### 2.4 Data Analysis

Quantification of targeted compounds was performed using TargetLynx software, with peak areas calculated within a retention time window of ±15 s. The concentration calculations were estimated using a single‐point internal standard calibration approach. The data was imported into MetaboAnalyst 6.0 for multivariate analysis, applying principal component analysis (PCA) and orthogonal projections to latent structures‐discriminate analysis (OPLS‐DA). Lipids with a variable importance in projection (VIP) greater than 1 and *t*‐test *p* values less than 0.05 were identified as significantly different. Hierarchical clustering heatmaps were generated to visually illustrate differences between groups.

### 2.5 Statistical Methods

Data were expressed as mean ± standard error of the mean (S.E.M.), and analyzed using GraphPad Prism 8 (GraphPad Software Inc., San Diego, USA). Statistical comparisons across groups were performed by one‐way analysis of variance (ANOVA), followed by Dunnett's post hoc tests for multiple comparisons. All reported *p* values were two‐tailed, and statistical significance was defined at *α* = 0.05 level.

## 3 Results

### 3.1 Effect of ACPA or AM1241 on ASD‐Like Behavioral Phenotypes

**FIGURE 1:**
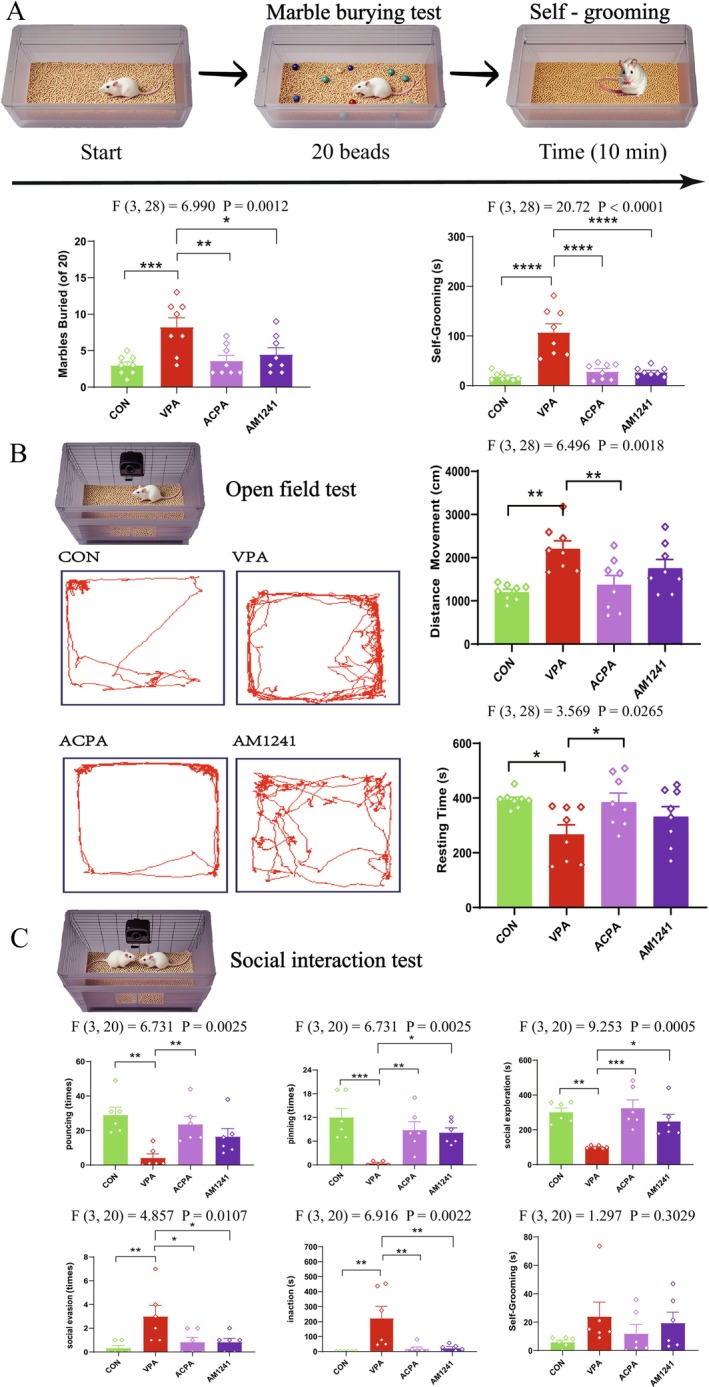
Effect of ACPA and AM1241 on ASD‐like behavioral phenotypes in VPA‐exposed rats. (A) Marble burying test and self‐grooming test. (B) Open field test. (C) Social interaction test. Data represented as mean ± SEM (*n* = 6–8 per group). Results were analyzed by one‐way ANOVA with post hoc Dunnett's test (*p < 0.05, **p < 0.01, ***p < 0.001, ****p < 0.0001).

VPA‐exposed rats had a significant increase in the number of marbles buried and the duration of self‐grooming compared with the CON group (*p* < 0.001). Both ACPA and AM1241 intervention significantly reduced the number of marbles buried and the duration of self‐grooming time of VPA‐exposed rats (Figure 1A, *p* < 0.05). In the open field test (Figure 1B), VPA‐exposed rats displayed hyperactivity as demonstrated by greater total movement distance and shorter resting time than the CON rats (*p* < 0.05). After ACPA intervention, the total movement distance of VPA‐exposed rats was significantly reduced, and the resting time increased (*p* < 0.05). While, there was no significant difference between the VPA group and the AM1241 group. As shown in Figure 1C, VPA‐exposed rats exhibited a lower level of social behaviors (pouncing, pinning, social exploration) and a higher level of nonsocial behaviors (social evasion, inaction) in the social interaction test (*p* < 0.01). Injected with ACPA increased pouncing, pinning, and social exploration, and decreased social avoidance and inaction (*p* < 0.05). Similarly, AM1241 injection increased pinning and social exploration and decreased social avoidance and inaction (*p* < 0.05). There was no significant difference in self‐grooming between the four groups.

Collectively, these findings suggest that VPA‐exposed rats display characteristics that effectively model the primary behavioral symptoms associated with ASD. Furthermore, activation of either CB1R or CB2R appears to alleviate the increased stereotyped behaviors, hyperactivity, and impaired social interactions observed in these VPA‐exposed rats (Figure 1).

### 3.2 ACPA and AM1241 Ameliorated the Abnormal Lipid Metabolism in VPA Rats

**FIGURE 2:**
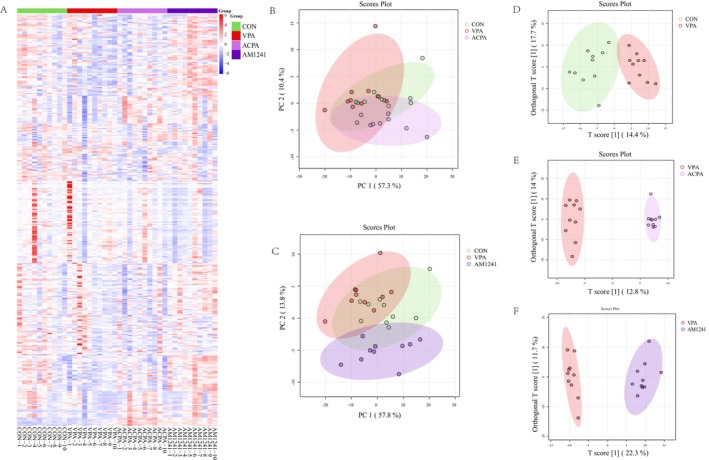
Effect of ACPA and AM1241 on lipid metabolism in VPA‐exposed rats. (A) Cluster heat map of lipid metabolite expression levels (*n* = 10 per group). (B, C) PCA score plots of hippocampal lipidomics. (D, E, F) OPLS‐DA score plots CON versus VPA group, VPA versus ACPA group, and VPA versus AM1241 group.

The hierarchical clustering heatmap illustrated distinct expression patterns of lipid profiles in the hippocampus across the four experimental groups: CON, VPA, ACPA, and AM1241 (Figure 2A). The clustering pattern suggested that VPA exposure significantly altered lipid metabolism compared to the CON group, while interventions with ACPA and AM1241 induced separate but noticeable changes in lipid profiles compared to the VPA group. As depicted in Figure 2B,C, PCA score plots showed that the CON, VPA, and treated groups could not be clearly distinguished. To further classification, a supervised OPLS‐DA model was employed, revealing a clear separation between the CON and VPA groups, as well as between the VPA and ACPA group, and the VPA and AM1241 groups (Figure 2D–F), confirming that both interventions significantly altered the lipidomic landscape.

**FIGURE 3:**
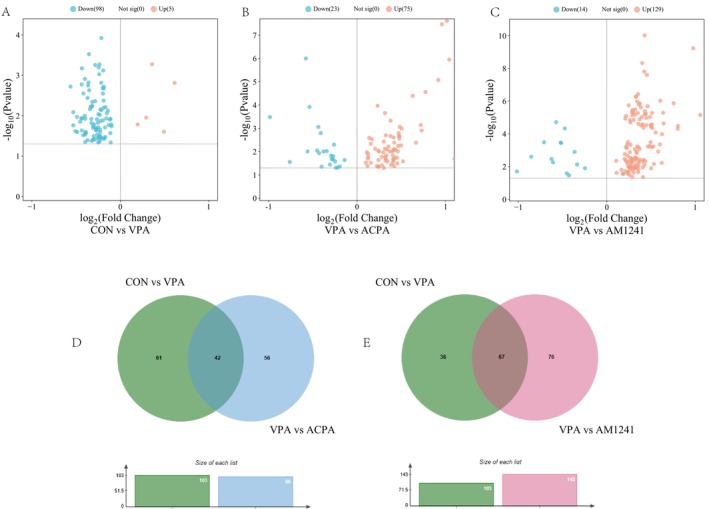
Two groups of samples difference lipid metabolites were analyzed. (A) CON group and VPA group difference lipid metabolites expression in the volcanic figure; (B) Volcano diagram of differential lipid metabolites expression between the VPA group and the ACPA group; (C) VPA group and AM1241 group of different lipid metabolites expression volcano diagram; (D, E) Venn diagram of difference lipid metabolites.

Based on the OPLS‐DA model, differential lipid metabolites were identified according to the selection criteria of VIP > 1 and *p* < 0.05. The results revealed that 98 lipid metabolites were significantly downregulated in the VPA group compared to the CON group, including phosphatidylcholine (PCs), lysophosphatidylcholine (LPCs), LPC O‐ (16:0, 18:0, 18:1), fatty acids (FAs), sphingomyelin [SM (35:0, 37:2, 42:4)], ceramide [(Cer) d16:1/22:0, d18:1/23:1], and phosphatidylethanolamine [PE (38:2, 40:3)]. While 5 lipid metabolites were upregulated, including FA (21:0, 26:0, 28:0), PC O‐30:0, and SM 34:1 (Figure 3A, Table S1). Compared to the VPA group, APCA intervention led to the upregulation of 75 lipid metabolites and downregulation of 23, with 42 common differential lipid metabolites were found (Figure 3B,D, Table S2). Table 1 presented 39 lipid metabolites that counter‐regulated by ACPA in VPA‐exposed rats, including upregulation of PCs, LPC (18:3, 22:1), FAs, SM (37:2, 42:4), Cer (d16:1/22:0, d18:1/23:1), while downregulation of PC O‐30:0 and SM 34:1. Similarly, AM1241 intervention resulted in 129 upregulated lipid metabolites and 14 downregulated ones compared to the VPA group, with 67 common differential lipid metabolites were identified (Figure 3C,E, Table S3). Table 2 showed 64 lipid metabolites that were counter‐regulated by AM1241 in VPA‐exposed rats, including upregulation of PCs, LPCs, LPC O‐ (16:0, 18:0, 18:1), FA (22:3, 22:4), SM (35:0, 42:4) and PE (38:2, 40:3), while downregulation of SM 34:1.

**TABLE 1:** ACPA regulates differential lipid metabolites expression in VPA‐exposed rats.

Lipids	Trend of expression		Lipids	Trend of expression	
	VPA vs. CON	ACPA vs. VPA		VPA vs. CON	ACPA vs. VPA
PC 32:1	Down	Up	LPC 18:3	Down	Up
PC 36:1	Down	Up	LPC 22:1	Down	Up
PC 38:1	Down	Up	FA 14:1	Down	Up
PC 38:2	Down	Up	FA 16:0	Down	Up
PC 38:4	Down	Up	FA 16:1	Down	Up
PC 38:5	Down	Up	FA 18:1	Down	Up
PC 39:2	Down	Up	FA 18:3 ALA	Down	Up
PC 40:1	Down	Up	FA 20:3	Down	Up
PC 40:2	Down	Up	FA 20:4 AA	Down	Up
PC 40:3	Down	Up	FA 20:5 EPA	Down	Up
PC 40:7	Down	Up	FA 22:3	Down	Up
PC 41:2	Down	Up	FA 22:4	Down	Up
PC 42:2	Down	Up	FA 22:5 DPA	Down	Up
PC 42:3	Down	Up	FA 22:6 DHA	Down	Up
PC 42:7	Down	Up	SM 34:1	Up	Down
PC 42:8	Down	Up	SM 37:2	Down	Up
PC 44:8	Down	Up	SM 42:4	Down	Up
PC 46:6	Down	Up	Cer (d16:1/22:0)	Down	Up
PC 46:7	Down	Up	Cer (d18:1/23:1)	Down	Up
PC O‐30:0	Up	Down			

Abbreviations: Cer, Ceramide; down, down regulation; FA, Fatty acids; LPC, Hemolysis phosphatidylcholine; PC, Phosphatidylcholine; SM, Sphingomyelin; up, up regulation.

**TABLE 2:** AM1241 regulates differential lipid metabolites expression in VPA rats.

Lipids	Trend of expression		Lipids	Trend of expression	
	VPA vs. CON	AM1241 vs. VPA		VPA vs. CON	AM1241 vs. VPA
PC 32:1	Down	Up	PC 42:8	Down	Up
PC 33:1	Down	Up	PC 44:8	Down	Up
PC 34:1	Down	Up	PC 44:9	Down	Up
PC 35:2	Down	Up	PC 46:6	Down	Up
PC 36:0	Down	Up	PC 46:7	Down	Up
PC 36:1	Down	Up	LPC 15:0	Down	Up
PC 36:4	Down	Up	LPC 16:1	Down	Up
PC 37:1	Down	Up	LPC 16:2	Down	Up
PC 37:3	Down	Up	LPC 17:0	Down	Up
PC 37:5	Down	Up	LPC 17:1	Down	Up
PC 38:0	Down	Up	LPC 17:2	Down	Up
PC 38:1	Down	Up	LPC 18:0	Down	Up
PC 38:2	Down	Up	LPC 18:1	Down	Up
PC 38:4	Down	Up	LPC 18:3	Down	Up
PC 38:5	Down	Up	LPC 19:1	Down	Up
PC 39:2	Down	Up	LPC 20:1	Down	Up
PC 39:5	Down	Up	LPC 20:2	Down	Up
PC 39:6	Down	Up	LPC 20:3	Down	Up
PC 40:1	Down	Up	LPC 22:1	Down	Up
PC 40:2	Down	Up	LPC 22:4	Down	Up
PC 40:3	Down	Up	LPC 22:5	Down	Up
PC 40:5	Down	Up	LPC O‐16:0	Down	Up
PC 40:6	Down	Up	LPC O‐18:0	Down	Up
PC 40:7	Down	Up	LPC O‐18:1	Down	Up
PC 41:2	Down	Up	FA 22:3	Down	Up
PC 41:7	Down	Up	FA 22:4	Down	Up
PC 42:2	Down	Up	SM 34:1	Up	Down
PC 42:3	Down	Up	SM 35:0	Down	Up
PC 42:4	Down	Up	SM 42:4	Down	Up
PC 42:5	Down	Up	Cer (d18:1/23:1)	Down	Up
PC 42:6	Down	Up	PE 38:2	Down	Up
PC 42:7	Down	Up	PE 40:3	Down	Up

Abbreviations: Cer, Ceramide; down, down regulation; FA, Fatty acids; LPC, Hemolysis phosphatidyl choline; PC, Phosphatidyl choline; PE, Phosphatidylethanolamine; SM, Sphingomyelin; up, up regulation.

Overall, these findings suggest that VPA exposure significantly disrupted hippocampal lipid metabolism, whereas activations of CB1R or CB2R modulated these disruptions by restoring specific lipid metabolites.

## 4 Discussion

In this study, we explored how activation of CB1R and CB2R influences behaviors associated with ASD and lipid metabolic abnormalities in a VPA‐induced rat model. The findings revealed that treatment with the CB1R agonist ACPA and the CB2R agonist AM1241 significantly alleviated core behavioral symptoms of ASD, such as repetitive, stereotyped, and impaired social interaction. Additionally, these treatments corrected abnormal hippocampal lipid metabolism. Our study highlights the significant role of cannabinoid receptor signaling in the pathology of ASD and suggests their potential for therapeutic development.

Our results that VPA‐exposed rats exhibited core ASD‐like traits were in line with prior studies, supporting prenatal VPA exposure in rodents as a well‐established model inducing ASD‐like phenotypes. The administration of the CB1R agonist ACPA and the CB2R agonist AM1241 effectively reduced stereotyped behaviors and improved social deficits in VPA‐exposed rats. Lines of work have illustrated the importance of CB1R and CB2R in ASD‐relevant behavioral manifestations. Fyke et al. indicated that the full knockout of CB1R resulted in marked deficits in social interest and social investigation [17]. Moreover, the blockade of CB1R abolished the prosocial effect of increased ECS signaling in two widely studied ASD mouse models, BTBR mice and VPA‐exposed offspring [18, 19]. However, an opposite outcome was observed when intra‐amygdala administration of a CB1R antagonist improved the impaired sociability in Fmr1Δexon8 rats [20]. Ettaro et al. found that acute CB1R antagonist administration reduced locomotor activity, increased scratching, and grooming, while chronic high‐dose administration improved marble burying behavior and social interaction [21]. These bidirectional effects suggested that the impact of CB1R on ASD‐like behaviors might depend on dosage, administration method, and affected neural circuits. Interestingly, some researchers support the use of the CB1R full knockout mouse in preclinical research related to ASD [17, 22]. CB2R is located in glial cells and also in neurons, and its role in pathological neurophysiology being an interesting field. It was found that the knockout of CB2R in neurons increased stereotyped behaviors, hyperactivity, and reduced social interactions in mice [23]. There was also an impairment in social memory in CB2R deletion mice of both sexes [24]. In addition, selective CB2R agonist treatment improved depressive‐like and stereotyped behaviors, while these effects were blocked by a CB2R antagonist [25]. Combined with our findings, there results understored that activation of the CB1R and CB2R signaling pathways is involved in regulating ASD‐like behaviors, and their activation could ameliorate ASD symptoms in VPA‐induced mice.

Given the significance of the hippocampus as a critical brain area for understanding ASD, further study of this region is particularly important [26], we conducted targeted lipidomics analysis on the hippocampal tissue. Our data revealed that VPA exposure induced significant lipid metabolism alterations in the hippocampus, characterized by downregulation of phosphatidylcholines (PCs), lysophosphatidylcholines (LPCs), fatty acids (FAs), sphingomyelins (SMs), ceramides (Cers), and phosphatidylethanolamines (PEs), alongside increases in specific FAs (21:0, 26:0, 28:0), PC O‐30:0, and SM 34:1. Notably, CB1R and CB2R activation significantly reversed these VPA‐induced lipid alterations. Treatment with ACPA restored the levels of PCs, LPCs, FAs, SMs, and Cers, while reducing PC O‐30:0 and SM 34:1, suggesting a therapeutic effect on ASD‐related lipid abnormalities. AM1241 treatment exhibited a similar corrective pattern. Our previous study identified abnormal polyunsaturated fatty acids (PUFAs) and SM metabolism in children with ASD, highlighting docosahexaenoic acid (DHA) and sphingosine 1‐phosphate (S1P) as significant predictors of ASD [6]. It has been well acknowledged that decreased levels of PUFAs in children with ASD [8]. Here, we found activation of CB1R reversed the lower PUFAs in VPA‐exposed offspring, particularly arachidonic acid (FA 20:4), eicosapentaenoic acid (FA 20:5), and DHA (FA 22:6), essential for neurodevelopment. Furthermore, PCs and LPCs, key brain lipid metabolites involved in neurotransmission, inflammation, and oxidative stress, were notably decreased in the ASD model, aligning with prior research [27, 28]. Importantly, activation of CB1R or CB2R upregulated the lower levels of PCs and LPCs. There was evidence that blocking CB1R promoted fatty acid oxidation and mitochondrial biogenesis enzymes and downregulated lipogenesis [29, 30]. CB1R activation increased lipogenic gene expression, and its overactivation was linked to metabolic syndrome [31]. These studies pointed out that the inhibition of CB1R could ameliorate lipid metabolism‐related disorders. In contrast, the present study revealed CB1R activation restored aberrant lipid metabolism in ASD model rats, indicating an ongoing debate about CB1R's regulatory role in lipid metabolism. Functional studies also indicated opposing effects of CB1R and CB2R, with CB2R activation consistently showing positive lipid metabolic outcomes, such as preventing obesity, nonalcoholic fatty liver disease, and diabetes [32, 33, 34]. Our findings support this beneficial role of CB2R activation. A recent study in immunometabolism has provided new insight that the transitions of microglial phenotype and function to adapt inflammatory responses were mediated by reprogramming of metabolic pathways. Specifically, microglial lipid metabolism, including synthesis and oxidation, has been implicated in brain function in disorders characterized by neuroinflammation [35]. It is well known that CB2R is highly expressed in activated microglia under neuroinflammatory conditions, and our previous research has revealed its upregulation in ASD [12]. Collectively, we hypothesized that the beneficial effects of CB2R activation on lipid metabolism and ASD‐like behaviors may be mediated, at least in part, through its regulation of microglial neuroinflammatory responses. However, the underlying mechanisms by which CB1R and CB2R modulate lipid metabolism require further exploration.

## 5 Limitations

Some limitations of this study should be acknowledged. First, the administration of CB1R and CB2R agonists via intraperitoneal injection may introduce systemic effects, making it challenging to distinguish central nervous system‐specific actions from peripheral influences. Second, this study would have been strengthened by evaluating the potential effects of cannabinoid receptor agonists in control animals. Clarifying whether the observed effects are specific to the pathological condition is essential. Third, given that ASD is a multifaceted disorder influenced by genetic, epigenetic, and proteomic factors, further study should integrate multiomics approaches to comprehensively elucidate the underlying molecular mechanisms linking cannabinoid receptor signaling, lipid metabolism, and ASD pathology.

## 6 Conclusions

This study provides new evidence linking ASD‐like behaviors, lipid metabolism abnormalities, and endocannabinoid system regulation. Our results demonstrated that CB1R and CB2R activation alleviated VPA‐induced ASD‐like behaviors and restored disrupted lipid profiles in the hippocampus, suggesting a potential therapeutic approach for ASD. Further research should explore the molecular mechanisms underlying CB1R‐ and CB2R‐mediated lipid regulation and their implications for ASD treatment strategies.

## Ethics Statement

All experiments were approved by the Ethics Committee of Harbin Medical University (HMUIRB2020007).

## Consent

All authors reviewed the manuscript and agreed to publish it.

## Conflicts of Interest

The authors declare no conflicts of interest.

## Supporting information


**Data S1:** cns70591‐sup‐0001‐DataS1.xlsx.

## Data Availability

The data that support the findings of this study are available from the corresponding author upon reasonable request.
